# Expectant management for umbilical artery thrombosis in the third trimester of pregnancy: a case report

**DOI:** 10.3389/fphar.2024.1395344

**Published:** 2024-05-13

**Authors:** Mei-Qin Gong, Yong-Qing Zhang, Xiao-Dong Wang

**Affiliations:** ^1^ Department of Obstetrics and Gynecology, West China Second University Hospital, Sichuan University, Chengdu, China; ^2^ Key Laboratory of Birth Defects and Related Diseases of Women and Children, Sichuan University, Ministry of Education, Chengdu, China; ^3^ School of Computer Science, Chengdu University of Information Technology, Chengdu, China

**Keywords:** umbilical artery thrombosis, ultrasound monitoring, third trimester, fetal distress, case report

## Abstract

**Background:**

Umbilical artery thrombosis (UAT) is a rare complication of pregnancy and is associated with adverse pregnancy outcomes, including fetal intrauterine distress, intrauterine growth restriction, and still birth. UAT is unpredictable, and prenatal diagnosis is challenging. There is no consensus on the treatment strategy of UAT, especially for patients with prenatal detection of one of the umbilical artery embolisms. In most previous cases, an emergency cesarean section was performed, or intrauterine fetal death occurred at the time of UAT diagnosis.

**Case presentation:**

In this report, we describe a case of thrombosis in one of the umbilical arteries detected by routine ultrasonography at 31^+3^ weeks of gestation in a 34-year-old woman. Following expectant management with intensive monitoring for 4 four days, an emergency cesarean section was performed because of abnormal fetal umbilical cord blood flow and middle cerebral artery blood flow; the newborn was in good condition at birth. The final umbilical cord histopathology revealed thrombosis in one of the umbilical arteries. Both mother and newborn described in this case underwent long-term follow-up for nearly 2 two years and are currently in good health without any complications.

**Conclusions:**

Based on our experience, obstetricians should comprehensively consider the current gestational age and fetal intrauterine status when UAT is suspected to determine the best delivery time. The appropriate gestational age should be prolonged as long as the mother and fetus are stable when the fetus is immature, trying our best to complete the corticosteroid treatment to promote fetal lung maturity and magnesium sulfate to protect fetal brain. During expectant management, ultrasound monitoring, electronic fetal heart monitoring, and fetal movement counting should be strengthened. Clinicians should ensure that the patients and their families are informed about all potential risks of expectant management for UAT.

## Background

UAT is a rare obstetric complication that can lead to severe adverse pregnancy outcomes such as fetal distress, fetal growth restriction, and fetal death, mainly when UAT occurs during the first half of gestation (Heifetz, 1988; Sato and Benirschke, 2006). The reported incidence of UAT ranges from 0.0025% to 0.045% of gestation (Kitano et al., 2018). The occurrence of umbilical arterial thrombosis is unpredictable. Most fetuses with UAT have no specific clinical signs or symptoms; the common clinical manifestations are abnormal fetal movement, fetal heart rate monitoring, fetal growth restriction, etc (Li et al., 2019a). Prenatal diagnosis of UAT mainly depends on accurate ultrasound scanning. Another question that confuses clinicians is how to deal with umbilical artery embolism that is suspected on prenatal ultrasound scans. Still, it is tough to decide whether to perform expectant management, especially for fetuses with a low gestational age. Few cases of expectant management have been reported in the literature. Here, we report a case of UAT diagnosed by ultrasound examination, and expectant management was performed under close monitoring; moreover, promoting fetal lung maturity with corticosteroids and protecting fetal brain with magnesium sulfate was finished. Finally, the mother and the child had a good outcome after nearly 2 years of follow-up.

## Case presentation

**FIGURE 1:**
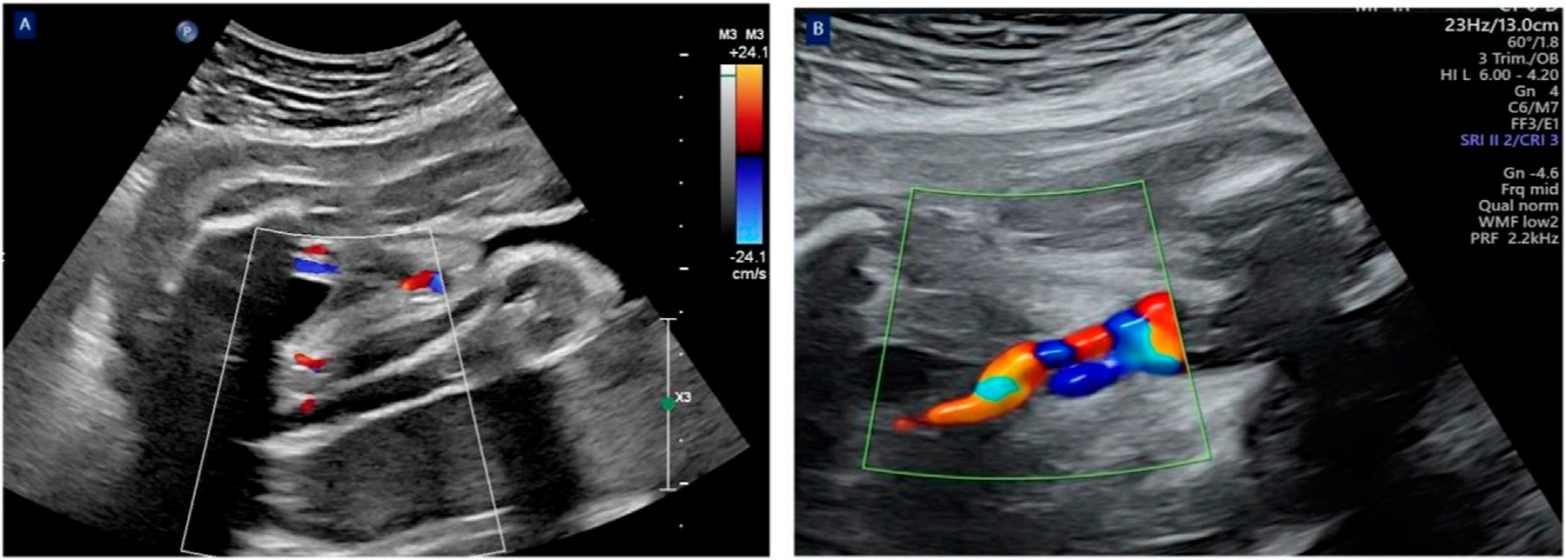
The cross-sectional view of the umbilical cord revealed two umbilical arteries with blood flow signals and one venous blood flow signal at 31 weeks of gestation **(A)**. The cross-sectional view of the umbilical cord revealed only one umbilical artery with a blood flow signal and one venous blood flow signal at 31+3 weeks of gestation **(B)**.

A 34-year-old multipara (gestation, 5; parity,1; abortion, 3) who had spontaneously conceived presented to our hospital for routine prenatal ultrasound scanning. She was admitted to the hospital due to threatened preterm birth at 31 weeks. This patient had undergone induced abortions twice at about 8 weeks of gestation, 5 and 8 years previously, and a biochemical pregnancy 2 years earlier. She had undergone a laparoscopic cholecystectomy 21 years ago and a term cesarean section because of abnormal fetal heart monitoring 7 years ago, and she had no history of obstetrical antiphospholipid syndrome, which commonly leads to arterial and venous thrombosis. The anti-β2Glycoprotein I (anti-β2GPI) and anticardiolipin (anti-CL) antibodies were positive at 11+2 weeks. Multiple dynamic reexaminations during pregnancy were still positive (the interval was more than 12 weeks), the diagnosis of non-criteria obstetric antiphospholipid syndrome was considered by rheumatologist. The patient was treated with multiple medications according to the rheumatologist at 23+4 weeks, including 6000 iu low molecular heparin calcium injected subcutaneously daily until admission for termination of pregnancy, aspirin 100 mg orally daily until 36 weeks gestation, methylprednisolone tablets 4 mg orally daily until admission for termination, and hydroxychloroquine sulfate 200 mg orally twice daily until admission for termination, blood coagulation and liver function were dynamically monitored during the treatment. At 12^+2^ weeks, ultrasound scanning showed a nuchal translucency thickness of 1.2 mm. At 24 weeks, two umbilical arteries and one umbilical vein were detected by conventional color Doppler ultrasound (Figure 1). The testing of cell-free fetal DNA in the maternal peripheral blood was negative, and no structural anomalies were observed. The fetal weight was 1,337 g estimated by ultrasound to be 3.9% of its same gestational age weight when the patient was admission at 31 weeks. Therefore, the diagnosis of fetal growth restriction was considered.

The patient was admitted due to threatened preterm birth at 31 weeks. Magnesium sulfate was given to protect fetal brain, and corticosteroids were shown to accelerate fetal lung maturity after admission. A routine ultrasound scanning during hospitalization revealed a single umbilical artery at 31^+3^ weeks (Figure 1); umbilical artery thrombosis was considered. Expectant management was processed to prolong the gestational age as much as possible to improve the adverse pregnancy outcomes and to avoid highly preterm infant birth after informing all potential risks of expectant management for UAT patients and their families. Our expectant management included four components: (1) medications were continued as directed by the rheumatologist. (2) daily intermittent oxygen inhalation (twice daily for 30 min) and intensive monitoring, including fetal heart rate auscultation every 4 h, electronic fetal heart rate monitoring twice daily, and informing the patient to count fetal movements three times daily. (3) Daily ultrasound screening of the umbilical artery, middle cerebral artery, and ductus venous blood flow, mainly calculating the cerebroplacental ratio. (4) assessment of the patient’s risk of thrombosis: Compared with women with a history of thrombosis, recurrent miscarriage, or cessation of fetal development, women with persistently positive anti-β2Glycoprotein I (anti-β2GPI) and anticardiolipin (anti-CL) antibodies have a small risk of VTE (Royal College of Obstetricians and Gynaecologists, 2015). A British study found that persistent positive antiphospholipid antibodies did not affect pregnancy outcomes, but most scholars believe that persistent positive antiphospholipid antibodies can be seen as a risk factor for thrombosis (Soh et al., 2013). Therefore, we believe that this patient had risk factor for thrombosis.

**FIGURE 2:**
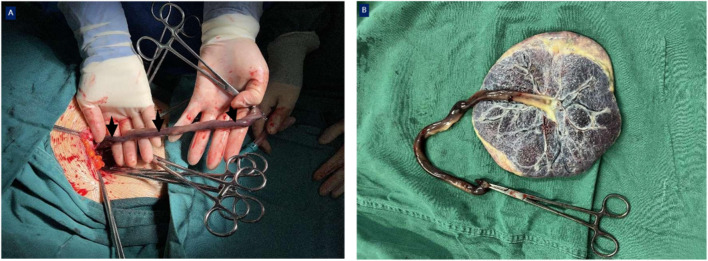
Intraoperative gross view. AT (black arrows) was observed in this fetus; the umbilical cord was dull red in color and rigid **(A)**, inserted into the center of the placenta **(B)**.

**FIGURE 3:**
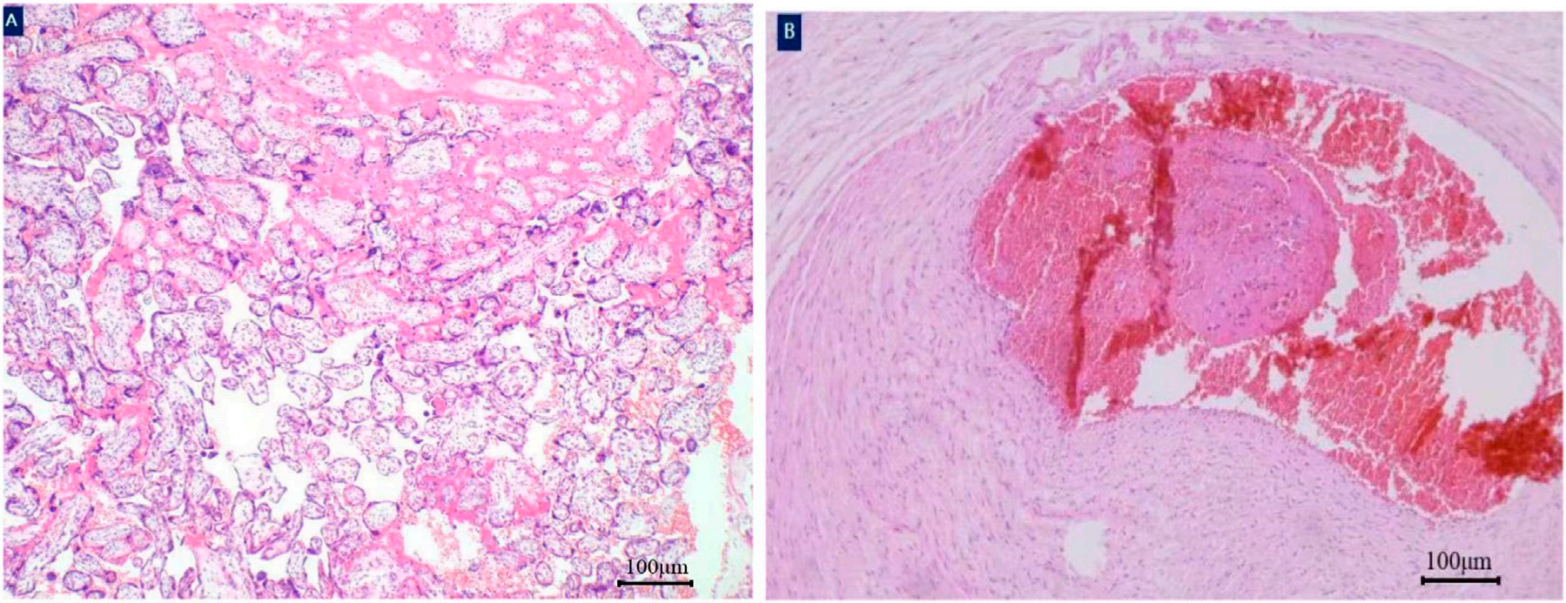
Histology of the umbilical cord. A white thrombus was found in the umbilical artery of fetus. Placenta parenchyma hematoxylin and eosin×40 magnification **(A)**. Thrombosis of umbilical artery hematoxylin and eosin×40 magnification **(B)**.

Four days later, the umbilical artery pulsatility index and middle cerebral artery pulsatility index were significantly decreased, and both were distinctly lower than the 5th percentile of the same gestational age. The patient and her family were fully informed of the risks of continuing pregnancy, and an emergency cesarean section was performed after they signed related medical documents. A girl weighing 1480 g was delivered, with 1-5-10 min Apgar scores of 9–10-10, and the amniotic fluid was clear. The length of the umbilical cord was approximately 45 cm; gross examination revealed one side of the umbilical cord was dull red in color and appeared rigid (Figure 2). The cut surface showed one vein and two arteries in the umbilical cord, with one of the arteries displaying blood flow blockage. Finally, the pathological examination of the placenta also confirmed a thrombus in one of the umbilical arteries (Figure 3).

Thrombosis of umbilical artery hematoxylin and eosin×40 magnification (B); Due to prematurity, the newborn was transferred to the neonatal intensive care unit and required respiratory support. Thankfully, the infant experienced favorable postnatal development without any serious complications and was discharged from our hospital on the 23rd day of life, weighing 1,670 g. The mother was also discharged successfully on the third day after surgery. Close follow-ups have proceeded for nearly 2 years; until now, the mother is in good health, and the child has no abnormalities in the physical, mental, or intellectual development.

## Discussion and conclusion

Umbilical cord thrombosis has been reported to occur in approximately 1 out of 1,300 deliveries, 1 out of 1,000 postnatal autopsies, and 1 out of 250 high-risk pregnancies (Heifetz, 1988). The specific pathogenesis of umbilical artery thrombosis is still unclear; it may be related to excessive umbilical cord length, true cord knot, umbilical cord anatomic abnormalities such as torsion, vascular mechanical injury and primary or secondary maternal-fetal thrombosis (Royal College of Obstetricians and Gynaecologists, 2015). Umbilical cord blood embolism can block umbilical cord blood flow, resulting in fetal hypoxia, intrauterine growth restriction, and even intrauterine death; in this case, fetal growth restriction was present. The etiology of cord vessel thrombosis may be explained by Virchow’s hypothesis, such as hypercoagulability, blood flow stasis, and endothelial injury. Hypercoagulability may be associated with maternal genetic or acquired factors. In this case, the high-risk factor was the combination of non-criteria obstetric antiphospholipid syndrome, the anti-β2Glycoprotein I, and anticardiolipin antibodies were strongly positive, and medical treatment was maintained throughout the pregnancy. Antiphospholipid syndrome (APS) is a non-inflammatory autoimmune disease characterized by arterial and venous thrombosis, pathological pregnancy (including abortion in the first trimester and stillbirth in the second and third trimester), and thrombocytopenia. Antiphospholipid antibodies mainly include lupus anticoagulant, anti-β2Glycoprotein I, and anticardiolipin antibodies (Soh et al., 2013); aPL can mediate vascular endothelial anticoagulant dysfunction and dysregulation of complement activation regulation mechanism, resulting in thrombophilia (Qian and Zhen, 2019). In addition, previous studies have reported that diabetes history, smoking history, and obesity were risk factors for umbilical cord thrombosis (Jieqiong and Wen, 2018; Guocai et al., 2019).

Ultrasonography is the preferred method for prenatal detection of umbilical cord abnormalities. In recent years, clinicians have attached great importance to the role of umbilical cord factors in perinatal adverse outcomes. The two-dimensional ultrasound transverse section of the normal umbilical cord shows three ring structures of “one big ring and two small rings”; one big ring is an umbilical vein, and two small rings are the umbilical artery. According to the direction of blood flow and probe, color ultrasound examination shows that the umbilical cord blood flow is “red, blue, blue” or “blue, red, red”; three blood vessels are arranged in a spiral. Umbilical artery embolism usually occurs in the third trimester of pregnancy, and umbilical artery embolism should be differentiated from the single umbilical artery. When ultrasound shows hypoechoic or hyperechoic filling in the lumen of the umbilical artery, while previous ultrasound examination suggested a double umbilical artery, umbilical artery embolism could be diagnosed (Avagliano et al., 2010). The last ultrasound showed three umbilical vessels, one umbilical vein, and two umbilical arteries. At 31+3 weeks of gestation, a single umbilical artery was detected by reexamination of color ultrasound during hospitalization; umbilical artery thrombosis was considered; moreover, our final pathological diagnosis was umbilical arterial thrombosis. Highly experienced sonographers and obstetricians are required to make an accurate prenatal UAT diagnosis. Eventually, a pathological examination is needed to confirm UAT.

At present, there is no consensus on UAT’s treatment strategy. Most patients with prenatally diagnosed UAT choose to terminate their pregnancy by cesarean section within a short period after diagnosis. When UAT accompanied by fetal growth restriction is detected in the second trimester or early term, pregnancy management becomes challenging (Klaritsch et al., 2008). Obstetricians may be hesitant to intervene when dealing with a fetus and often rely on their own experience and fetal status to determine treatment options (Wu et al., 2016). In the study by Wang et al., it was demonstrated that it was worth considering expectant management treatment with low molecular weight heparin for patients with UAT due to apparent effects on the prolongation of gestational age. However, this study has several limitations. First, it was a small sample size analysis; only ten pregnant women with thrombosis of the umbilical cord were included. Second, all the reported cases were in the third trimester, so the effects of thrombosis on the fetus in the first or second trimester are unknown. Third, the long-term follow-up of neonates is inadequate. Thus, we could not evaluate survival rates and long-term implications of babies born with umbilical cord thrombosis (Wei et al., 2021). The particularity of our case was the combination of non-criteria obstetric antiphospholipid syndrome, we treated with low molecular weight heparin, but the umbilical artery thrombosis still occurred. Therefore, in the expectant management treatment of this patient, an increase in the dose of low molecular weight heparin may be considered to be beneficial in prolonging the gestational age, which needs to be further confirmed in subsequent studies. In the study by Wu et al. (Wu et al., 2022), it was found that the expectant management group did not have worse fetal outcomes compared to the urgent treatment group when current screening and therapeutic strategies were implemented (Li et al., 2019b), thirty-five pregnant women with thrombosis of umbilical cord were included in these analyses. Sometimes, sudden intrauterine fetal death due to umbilical cord thrombosis is unpredictable. Dussaux reported (Dussaux et al., 2014) a case at 32 weeks of gestation in which an ultrasound examination indicated local dilation of the umbilical vein lumen due to partial thrombosis, and intervention was not performed because the pregnant woman had no abnormalities in other indicators; 3 days later, the patient was admitted to the hospital due to reduced fetal movement and intrauterine fetal death was found (Wang et al., 2023). To date, there is controversy regarding how often the examination should be performed to determine the best term for termination of pregnancy before the onset of fetal distress or death by UAT, especially in the case of early preterm.

In our experience, expectant management requires intensive clinical parameter monitoring, especially ultrasound indices, fetal movement, electronic fetal heart monitoring, and coagulation-related changes. The most obvious change in our case was that both the umbilical artery pulse index and the middle cerebral artery pulse index were significantly reduced, and both were significantly lower than the 5th percentile for the same gestational age, while there were no obvious abnormalities in fetal movement, electronic fetal heart monitoring, and coagulation-related examinations. The patients and their families should be informed of all potential risks of expectant treatment and should write informed consent. Because of dynamic monitoring and timely intervention, our case had a good prognosis without complications. Clinicians should not only consider the adverse outcomes of preterm birth but also avoid unnecessary fetal loss. Ultrasound should be performed in patients with high-risk factors no more than 3 days despite normal fetal movement and heart monitoring. It is very urgent to complete magnesium sulfate to protect fetal brain and corticosteroids to accelerate fetal lung maturation. For medical institutions with the level of treatment of premature infants, we are more inclined to choose protective and expectant management patterns for fetuses with a gestational age of less than 32 weeks, except for other abnormalities. In contrast, fetuses with a gestational age greater than 32 weeks are categorized into the urgent termination of pregnancy. In addition, we recommend that the patients should be hospitalized for expectant management; emergency cesarean section can be performed immediately when we encounter signs of fetal distress, thereby avoiding adverse perinatal outcomes such as stillbirth.

## Data Availability

The original contributions presented in the study are included in the article/Supplementary Material, further inquiries can be directed to the corresponding author.
